# *In vivo* metabolic imaging of Traumatic Brain Injury

**DOI:** 10.1038/s41598-017-17758-4

**Published:** 2017-12-13

**Authors:** Caroline Guglielmetti, Austin Chou, Karen Krukowski, Chloe Najac, Xi Feng, Lara-Kirstie Riparip, Susanna Rosi, Myriam M. Chaumeil

**Affiliations:** 1 Department of Physical Therapy and Rehabilitation Science, University of California, San Francisco, CA USA; 2 Surbeck Laboratory of Advanced Imaging, Department of Radiology and Biomedical Imaging, University of California, San Francisco, CA United States; 3 Brain and Spinal Injury Center, University of California, 1001 Potrero Ave, Bldg. 1, Room 101, San Francisco, CA 94110 USA; 4 Department of Neurological Surgery, University of California, San Francisco, CA USA; 5 Weill Institute for Neuroscience, University of California, San Francisco, CA USA; 6 Kavli Institute of Fundamental Neuroscience, University of California, San Francisco, CA USA

## Abstract

Complex alterations in cerebral energetic metabolism arise after traumatic brain injury (TBI). To date, methods allowing for metabolic evaluation are highly invasive, limiting our understanding of metabolic impairments associated with TBI pathogenesis. We investigated whether ^13^C MRSI of hyperpolarized (HP) [1-^13^C] pyruvate, a non-invasive metabolic imaging method, could detect metabolic changes in controlled cortical injury (CCI) mice (n = 57). Our results show that HP [1-^13^C] lactate-to-pyruvate ratios were increased in the injured cortex at acute (12/24 hours) and sub-acute (7 days) time points after injury, in line with decreased pyruvate dehydrogenase (PDH) activity, suggesting impairment of the oxidative phosphorylation pathway. We then used the colony-stimulating factor-1 receptor inhibitor PLX5622 to deplete brain resident microglia prior to and after CCI, in order to confirm that modulations of HP [1-^13^C] lactate-to-pyruvate ratios were linked to microglial activation. Despite CCI, the HP [1-^13^C] lactate-to-pyruvate ratio at the injury cortex of microglia-depleted animals at 7 days post-injury remained unchanged compared to contralateral hemisphere, and PDH activity was not affected. Altogether, our results demonstrate that HP [1-^13^C] pyruvate has great potential for *in vivo* non-invasive detection of cerebral metabolism post-TBI, providing a new tool to monitor the effect of therapies targeting microglia/macrophages activation after TBI.

## Introduction

Traumatic brain injury (TBI) is a complex and heterogeneous brain pathology characterized by various degrees of tissue damage, hemorrhages and edema caused by the primary mechanical insult and subsequent secondary injury responses such as neuroinflammation^1–6^. Importantly, TBI is a major environmental risk factor for the development of neurodegenerative diseases with an estimated prevalence of 3.2–5.3 million Americans suffering from long-term complications and chronic disabilities^3,7^.

Impairment in energy metabolism following TBI has been reported to be associated with complex cellular processes in relation to disturbed ion homeostasis, mitochondrial defects, hypoxia, and inflammation^4–6,8,9^. Monitoring of brain metabolism after injury is of utmost importance, as it would help predict the incidence of secondary insults and define subsequent therapeutic strategies to minimize such occurrences^10^. To date, evaluation of cerebral metabolism usually relies on invasive methods, such as microdialysis, for direct assessment of brain metabolites or indirect measures of arterio-venous metabolite concentrations^11,12^. These methods have demonstrated that injured tissue is characterized by high lactate level and a high lactate-to-pyruvate ratio^13–15^. Amongst the non-invasive methods available in the clinical setting, proton magnetic resonance spectroscopy (^1^H-MRS) is of high interest as it allows the detection of brain metabolites, including lactate^16–18^. Several studies have shown that elevated lactate concentration in patients following brain injury correlates with poor prognosis, cognitive decline and high mortality^8,14,19,20^. However, because steady-state metabolites levels as detected by ^1^H-MRS do not directly reflect alterations in metabolic pathways, the exact mechanisms underlying the observed metabolic changes following TBI remain poorly understood and represent a matter of debate^8,21^.

Over the past decade, hyperpolarized ^13^C magnetic resonance spectroscopic imaging (HP ^13^C MRSI) has emerged as a clinically translatable neuroimaging method of high potential. This new imaging strategy allows monitoring of enzymatic reactions *in vivo* in real-time after injection of so-called hyperpolarized (HP) ^13^C-labeled probes^22–24^. HP ^13^C MRSI has notably improved the detection of cancerous lesions through the monitoring of increased conversion of HP [1-^13^C] pyruvate to HP [1-^13^C] lactate under aerobic conditions, the Warburg effect characteristic of cancer cells^25,26^.

Importantly, DeVience *et al*. have recently shown that HP ^13^C MRSI can detect altered HP [1-^13^C] pyruvate-to-lactate conversion in a rat model of TBI, at an acute time point (4 hours) following injury^27^. In an effort to assess the potential value of HP ^13^C MRSI in TBI, we here questioned whether HP ^13^C MRSI could monitor acute, subacute and chronic metabolic changes that occur following TBI. To do so, we performed a longitudinal study in mice that received a controlled cortical impact (CCI) and were imaged prior to injury (Baseline) and at acute (12 and 24 hours), subacute (7 days) and chronic (28 days) time points. Our results showed that HP [1-^13^C] lactate-to-pyruvate ratios were increased in the injured cortex at 12 hours, 24 hours, and 7 days post-injury. At these acute and sub acute time points, we additionally observed a decreased activity of pyruvate dehydrogenase (PDH), the enzyme which converts pyruvate into Acetyl-CoA, thus providing a likely mechanism for the observed increased HP [1-^13^C] lactate production.

We then evaluated whether HP ^13^C MRSI could detect microglia/macrophage activation which we have previously shown to contribute to secondary injury responses and cognitive decline following TBI^5,28^. We used the colony-stimulating factor-1 receptor (CSF1R) inhibitor PLX5622 to deplete up to 90% of the microglia in the adult mouse brain prior to CCI^29^. We found that microglia depletion prevented the increase in HP [1-^13^C] lactate-to-pyruvate ratios at 7 days post-injury, and that PDH activity was also unchanged compared to the contralateral hemisphere in PLX5622-treated brains. These results suggest a crucial role for microgliosis in energy metabolism changes at subacute time points following injury. Furthermore, our results highlight the potential of ^13^C MRSI of HP [1-^13^C] pyruvate to non-invasively detect alterations of cerebral metabolism after injury and provide a novel tool to monitor the effect of innovative therapies targeting microglia/macrophage activation after TBI.

## Materials and Methods

### Animals and experimental outline

All experiments were conducted in accordance with the National Institutes of Health Guide for the Care and Use of Laboratory Animals and were approved by the Institutional Animal Care and Use Committee of the University of California (San Francisco, CA).

Twelve to fifteen weeks old C57/BL6 male mice (n = 57 mice) were purchased from Jackson Laboratories and housed under 12:12 light-dark cycle with food and water ad-libitum. For longitudinal MR imaging, a subset of mice (n = 10 CCI; n = 5 Sham) were imaged prior to surgery (Baseline) and at twelve hours (12 hours), twenty-four hours (24 hours), seven days (7 days) and twenty-eight days (28 days) post-injury. A separate group of mice that did not undergo MR imaging was used for immunofluorescence staining and enzyme activity assays (n = 3–8 animals per time point). An additional group of mice (n = 11) received PLX5622 supplemented diet (Plexxikon Inc.) for a total period of fourteen days. After 7 days of receiving PLX5622 diet, all mice underwent CCI surgery and were kept to recover for an additional period of 7 days. A subset of these mice (n = 5) underwent MR imaging one day prior to CCI (Baseline) and 7 days post-injury. At 7 days post-injury, mice were euthanized and tissue was collected for immunofluorescence analyses (n = 5) and enzyme activity assays (n = 6). All mice were euthanized using a mixture of ketamine (150 mg/kg)/xylazine (15 mg/kg) in accordance with standard animal protocols. The time line for experiment is illustrated in Supplementary Fig. 1A-B, and the number of animals for each experimental group is detailed in Supplementary Tables 1 and 2.

### Surgical procedures

Animals were anesthetized and maintained under 2.5% isoflurane in O_2_ with a nonrebreathing nose cone and passive exhaust system connected to a stereotaxic frame (David Kopf). Once animals were secured with nontraumatic ear bars, eye ointment was applied and their heads were cleared of any hair around the scalp. After betadine application, a midline incision was made through the scalp. TBI was reproduced in the parietal lobe using the CCI model. Mice received a craniectomy ∼3.5 mm in diameter using an electric microdrill with the center point determined by a digitally calibrated manipulator arm (Leica) to the coordinates: anteroposterior, −2.0 mm; mediolateral, 2.0 mm, with respect to bregma. Explicit attention was paid to prevent damage to the dura during craniectomy; any animal in which the dura was disrupted, as assessed by excessive bleeding, was omitted from the study and replaced by another littermate. After craniectomy, contusion was achieved using a 3.0 mm convex tip attached to an electromagnetic impactor (Leica) mounted to the digitally calibrated manipulator arm. To impact flush with the natural curvature of the head/tissue, the manipulator arm was rotated 20° on the vertical axis. The parameters for impact were for a contusion depth of 0.95 mm (from dura), velocity was constant at 4.0 m/s, and the impact was sustained for 300 ms. After CCI injury, the scalp was sutured and each animal received 0.5 ml of physiologic saline (i.p.) before being placed in a water-heated incubation chamber (37 °C) until they fully recovered as exhibited by resumption of movement and grooming. Sham uninjured animals received the craniectomy as described above but the scalp was sutured withouth impact. All animals fully recovered from surgical procedures and exhibited normal weight gain for the duration.

### *In vivo* MR acquisitions

All *in vivo* MR experiments were conducted on a 14.1 tesla vertical MR system (Agilent Technologies, Palo-Alto, CA) equipped with 100 G/cm gradients and a dual tune ^1^H-^13^C volume coil (Ø_I_ = 40 mm) or a single tuned ^1^H proton coil (Ø_I_ = 40 mm). For each imaging session, mice were anesthetized using isoflurane (1–2% in O_2_) and a 27 G catheter was secured in the tail vein to allow for intravenous (iv) injection of the HP probe. Animals were then positioned in a dedicated craddle maintaining constant anesthesia and placed in the MR bore; respiration and temperature were continuously monitored during all acquisitions to ensure animal well-being and data reproducibility. First, axial T_2_-weighted images were acquired for adequate positioning of the grid used for HP ^13^C acquisitions. The following parameters were used: TE/TR = 20/1200 ms, slice thickness = 1.8 mm, number of averages = 2, matrix = 256 × 256, field of view (FOV) = 30 × 30 mm². For HP ^13^C MRSI acquisitions, 24 μL of [1-^13^C] pyruvate preparation was hyperpolarized using a Hypersense DNP polarizer (Oxford Instruments) for one hour^30^. After dissolution, HP [1-^13^C] pyruvate was rapidly dissolved in isotonic buffer (pH~7) to a final concentration of 80 mM. A final volume of 300 μL of the HP [1-^13^C] pyruvate solution was then injected iv over 12 sec through the tail vein catheter. From the beginning of the iv injection of HP [1-^13^C] pyruvate, 2D dynamic chemical shift imaging (CSI) ^13^C data were acquired using the following parameters: TE/TR = 1.2/60 ms; spectral width = 2500 Hz; 128points; 4 sec temporal resolution; flip angle (FA) = 10 deg; FOV = 24 × 24 mm²; 5 mm slice thickness. A representative dataset is shown in Supplementary Fig. 1C. Next, axial T_2_-weighted Fast Spin Echo images were acquired for the evaluation of CCI-induced brain lesions. The following parameters were used: TE/TR = 12/2000 ms, echo train = 8, slice thickness = 0.5 mm, number of averages = 2, matrix = 256 × 256, field of view (FOV) = 30 × 30 mm².

### MR data analysis

HP ^13^C MRSI datasets were analyzed using the in-house SIVIC software (http://sourceforge.net/apps/trac/sivic/) and custom-built programs written in MATLAB (MATLAB R2011b, The MathWorks Inc.). The k-space dimensions were zero-filled by a factor of two resulting in a 16 × 16 matrix. Spectra were summed over time and a lorentzian shape was used to fit the HP [1-^13^C] pyruvate and [1-^13^C] lactate peaks on the sum spectrum. Then, area under the curve (AUC) of HP [1-^13^C] pyruvate and AUC of HP [1-^13^C] lactate lorentzian fits were measured and the HP [1-^13^C] lactate-to-pyruvate ratio was calculated as the ratio of the AUC. Regions of interest including the injured and contralateral hemispheres were defined on T_2_-weighted images and HP [1-^13^C] lactate-to-pyruvate ratios were compiled, as described by Daniels *et al*.^31^, and reported as mean of the corresponding voxels for the injured and contralateral hemispheres. Color heatmaps of HP [1-^13^C] pyruvate, HP [1-^13^C] lactate and HP [1-^13^C] lactate-to-pyruvate ratio were generated using a linear-based interpolation of the ^13^C 2D CSI data to the resolution of the anatomical images using custom-built programs written in MATLAB and SIVIC. For anatomical analyses, three regions of interest including the ventricles, the lesioned area and the cavitation were manually delineated on T_2_-weighted images according to the Franklin and Paxinos anatomical mouse brain atlas with AMIRA software (Mercury Computer systems, San Diego, USA), as illustrated in Supplementary Fig. 2. Next, the volume of the regions of interest and 3D reconstruction were obtained using AMIRA software.

### Immunofluorescence acquisition and analysis

All immunofluorescence analyses were performed according to previously described procedures. First, mice were transcardially perfused with ice-cold phosphate buffered saline followed by ice-cold 4% paraformaldehyde (PFA). Next, brains were dissected and further fixated in 4% PFA for 2 h, then dehydrated through a sucrose gradient (2 hours at 5%, 2 hours at 10% and overnight at 20%). Afterwards, brain tissue was snap-frozen in liquid nitrogen and kept at −80 °C until further processing. Ten µm-thick cryosections were collected. Immunofluorescence staining was performed on brain slides using the following antibody combinations: a primary rabbit anti-Iba1 antibody (Wako, 019–19741, 1:500 dilution) with a secondary donkey anti-rabbit Alexa Fluor 555 (Invitrogen, A31572, 1:1000 dilution), a primary rat anti-CD68 (BioRad, MCA1957, 1:100 dilution) with a secondary anti-rat AF488 (Life Technologies, A11006, 1:200) and a primary rat anti-mouse CD11b (AbD Serotec, MCA711G, 1:200) with a secondary anti-rat AF488 (Life Technologies, A11006, 1:200). Slides were counterstained using Hoechst 33342 (Invitrogen, H3570, 1:2000 dilution). Following staining, sections were mounted using Prolong Gold Antifade (Invitrogen, P36930). All imaging was achieved using a Zeiss Imager.Z1 Apotome microscope controlled by ZEN software (Zeiss 2012). Quantitative analyses of immunofluorescence images were performed using NIH ImageJ analysis software (v1.46r). The degree of microglia/macrophages (Iba-1) and CD68 and CD11b expression was determined based on the image-covering staining and expressed as percentage of the total area, as previously described^32^.

### Spectrophotometric assays

Separate groups of mice underwent CCI surgery and were euthanized and transcardially perfused with ice-cold phosphate buffered saline at each time point of interest (Naive, 12 hours, 24 hours, 7 days and 28 days post-injury). Mice that received the PLX5622 or control diet were euthanized at 7 days post-injury. Brains were rapidly dissected and the ipsilateral to injury and contralateral cortices were isolated, snap-frozen and stored at −80 °C until further processing. The enzymatic activity of PDH was assessed using spectrophotometric activity assay kit (ab109902, Abcam), and the enzymatic activity of lactate dehydrogenase (LDH), (Vmax) was assessed as previously shown^33^. PDH and LDH enzymatic activities were normalized to the concentration of protein determined by the Bradford method; results are expressed as percentage of the contralateral cortices (n = 4–8 mice per group).

### Statistical analysis

Results are expressed as mean ± standard error of the mean (sem). A repeated measures ANOVA was used to evaluate statistical significance of the T_2_-weighted MRI. Two-Way ANOVA was used to determine statistical significance of HP ^13^C MRSI between injured and contralateral hemispheres over time. Statistical analyses of HP [1-^13^C] lactate-to-pyruvate ratio, expressed as a percent of the contralateral hemisphere, and enzyme activity assays for PDH and LDH were performed using a One-Way ANOVA. For immunofluorescence analyses, a t-test was used to evaluate statistical significance between control-treated and PLX5622-treated groups. All given p-values were corrected for multiple testing using the Tukey HSD post-hoc test (*p < 0.05, **p < 0.01, ***p < 0.001, ****p < 0.0001).

## Results

### Longitudinal evaluation of TBI using high field T_2_-weighted MR imaging

T_2_-weigthed MR images were acquired at high field strength (14.1 Tesla) to detect anatomical alterations following CCI. Mice were imaged prior to injury (Baseline), 12 hours, 24 hours, 7 days and 28 days post-injury.

**Figure 1:**
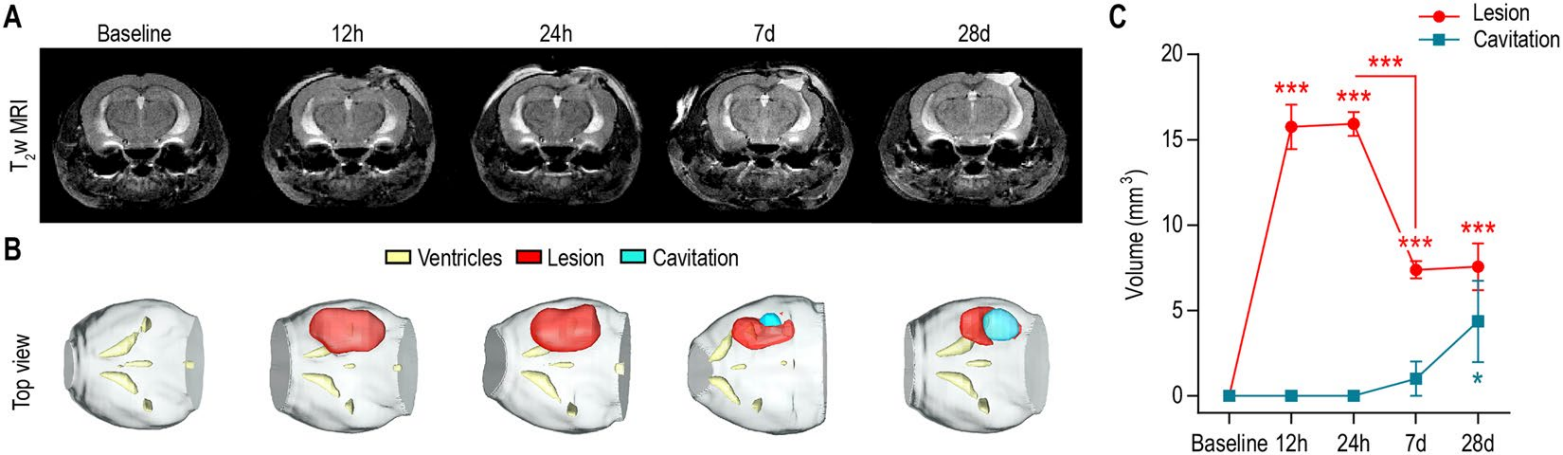
Longitudinal evaluation of TBI using high field T_2_-weighted MR imaging. (**A**) Representative T_2_-weigthed MR images were acquired prior to injury (Baseline), twelve hours (12 h), twenty-four hours (24 h), seven days (7 d) and twenty-eight days (28 d) post-injury. Cortical alterations of the tissue microstructure can be detected as a mix of hypo-intense and hyper-intense contrast in the injured hemisphere as early as 12 h post-injury, and persist until 28 d post-injury. The formation of a well-defined cavity can be observed at 7 d/28 d post-injury. (**B**) Representative 3D reconstruction of the lesioned area (red), cavitation (blue) and ventricles (yellow) for each time point. (**C**) Quantitative analyses revealed highest lesion size at acute time points following injury (12 h and 24 h, p < 0.0001, compared to Baseline). The lesioned area decreased by 53 ± 9% at 7 d (p = 0.0003, compared to 24 h) and persists until 28 d post-injury (p = 0.0005, compared to Baseline). The formation of a cavitation can be observed by 28 days post-injury (p = 0.0174, compared to Baseline). All values are reported as mean ± sem (n = 5 mice).

The lesioned area, composed of degenerating edematous tissue and blood, was detected as early as 12 hours post-injury and appeared as a mix of hyper-intense and hypo-intense contrast in the cortex of the hemisphere that received the CCI (Fig. 1A). Longitudinal T_2_-weigthed imaging showed that the volume of the lesioned tissue, depicted in red in the representative 3D reconstruction (Fig. 1B), peaks at 12 hours and 24 hours post-injury (Fig. 1C, p < 0.0001 at both 12 hours and 24 hours, compared to Baseline), decreases by 53 ± 9% at 7 days (p = 0.0003, compared to 24 hours), and persists until 28 days post-injury (p = 0.0005, compared to Baseline). At 7 days and 28 days post-injury, part of the lesioned tissue is being replaced by a well-defined cavity (p = 0.0174, compared to Baseline), most likely filled with cerebral spinal fluid, observed as a bright hyper-intense contrast on T_2_-weighted images (blue in the 3D reconstruction, Fig. 1B). In contrast, the total volume of the ventricles remained unchanged over time (p = 0.9292).

### Longitudinal MR metabolic imaging following TBI reveals a transient increase of the HP [1-^13^C] lactate-to-pyruvate ratio paralleled by decreased PDH activity

Next, we investigated whether ^13^C MRSI of HP [1-^13^C] pyruvate can detect metabolic alterations after CCI. To do so, we performed longitudinal ^13^C MRSI measurements prior to injury (Baseline) and at 12 hours, 24 hours, 7 days and 28 days post-injury.

**Figure 2:**
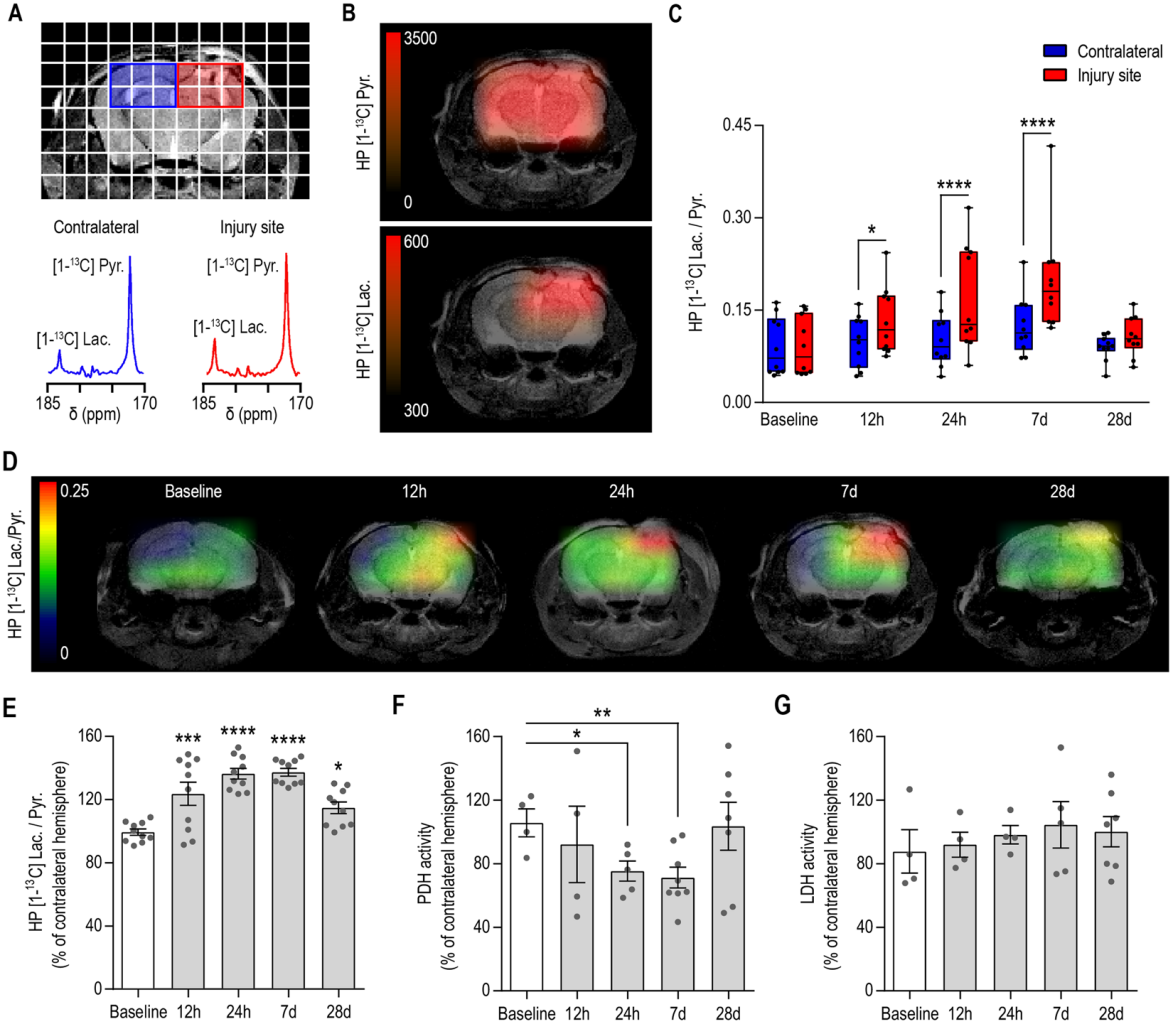
Longitudinal MR metabolic imaging following TBI reveals a transient increase of the HP [1-^13^C] lactate-to-pyruvate ratio, which is paralleled by decreased PDH activity. (**A**) Representative T_2_-weighted image with overlaid grid used for the acquisition of HP ^13^C MRSI, highlighting the voxels containing the injured (red) and contralateral (blue) hemispheres. HP ^13^C spectra for the contralateral hemisphere (blue) and injured hemisphere (red) showed increased HP [1-^13^C] lactate in the injured hemisphere at 7 days post-injury. (**B**) Corresponding heatmaps of HP [1-^13^C] pyruvate and HP [1-^13^C] lactate showed even distribution of HP [1-^13^C] pyruvate within the brain while HP [1-^13^C] lactate is increased at the level of the injury. (**C**) Quantitative analyses of the HP [1-^13^C] lactate-to-pyruvate ratios revealed a significant increase at 12 h, 24 h and 7 d post-injury between injured and contralateral hemispheres (Two-Way ANOVA, p < 0.0001 for hemisphere effect, p = 0.0259 for time effect, p = 0.0002 for hemisphere and time interaction). (**D**) Representative heatmaps indicating the highest level of the HP [1-^13^C] lactate-to-pyruvate ratio at the site of injury. (**E**) HP [1-^13^C] lactate-to-pyruvate ratio expressed as a percent change of the contralateral hemisphere showed increased ratios by 23 ± 7% at 12 hours (p = 0.0002), 36 ± 3% at 24 hours (p < 0.0001), 37 ± 2% at 7 days (p < 0.0001) and 15 ± 4% at 28 days post injury (p = 0.0132) compared to Baseline. (**F**) PDH activity was significantly decreased in the injured hemisphere by 30 ± 6% at 24 h (p = 0.0358) and 35 ± 7% at 7 d post-injury (p = 0.0011) while LDH activity remained unchanged (**G**). All values are reported as mean ± sem (n = 10 mice for HP [1-^13^C] MRSI, n = 4–8 mice per time point for PDH and LDH activity).

As shown in Fig. 2A, we observed an increase of HP [1-^13^C] lactate production in the HP ^13^C MR spectra from the injured hemisphere (red) compared to the contralateral hemisphere (blue) at 7 days post-injury. The corresponding heatmaps (Fig. 2B) show that HP [1-^13^C] pyruvate distributes evenly within the brain while HP [1-^13^C] lactate level is increased at the injured site.

Quantitative analyses presented in Fig. 2C revealed that HP [1-^13^C] lactate-to-pyruvate ratio was significantly different between injured and contralateral hemispheres (Two-Way ANOVA, p < 0.0001 for hemisphere effect, p = 0.0259 for time effect, p = 0.0002 for hemisphere and time interaction). Specifically, the HP [1-^13^C] lactate-to-pyruvate ratio of the injured hemisphere was significantly higher compared to the contralateral hemisphere at 12 hours (p = 0.0269), 24 hours (p < 0.0001) and 7 days (p < 0.0001) post-injury but not at 28 days post-injury (p = 0.5162). Corresponding HP [1-^13^C] lactate-to-pyruvate ratio heatmaps clearly indicate that the highest value of the HP [1-^13^C] lactate-to-pyruvate ratio was centered on the injury site (Fig. 2D). Next, we evaluated the percent increase of the HP [1-^13^C] lactate-to-pyruvate ratio in the injured hemisphere compared to the contralateral hemisphere. As shown in Fig. 2E, the HP [1-^13^C] lactate-to-pyruvate ratio in the injured hemisphere was significantly higher by 23 ± 7% at 12 hours (p = 0.0002), 36 ± 3% at 24 hours (p < 0.0001), 37 ± 2% at 7 days (p < 0.0001) and 15 ± 4% at 28 days post injury (p = 0.0132) compared to Baseline. As shown in Supplementary Fig. 3, the HP [1-^13^C] lactate-to-pyruvate ratios, expressed as percent change of the contralateral hemisphere, were significantly different between CCI and Sham animals (Two-Way ANOVA, p = 0.0001 for group effect, p < 0.0001 for time effect, p = 0.0702 for group and time interaction). Specifically, the HP [1-^13^C] lactate-to-pyruvate ratio of the CCI group was significantly higher compared to the Sham animals at 12 hours (p = 0.0456), 24 hours (p = 0.002) and 7 days (p = 0.0103) post-injury but not at 28 days post-injury (p = 0.2861). Additionally, whereas HP [1-^13^C] lactate-to-pyruvate ratios overtime showed a significant increase in the CCI group, at 12 hours (p = 0.0004), 24 hours (p < 0.0001) and 7 days (p < 0.0001) compared to Baseline, this ratio showed no significant difference at any time points in the Sham group (p ≥ 0.1004).

To further investigate the origin of HP [1-^13^C] lactate-to-pyruvate ratio alterations following injury, we evaluated the activity of two keys enzymes that control the fate of pyruvate, namely LDH, which converts pyruvate into lactate, and PDH which enables the entry of pyruvate into the TCA cycle. As shown in Fig. 2F,G, PDH activity was significantly decreased in the injured hemisphere by 30 ± 6% at 24 hours (p = 0.0358) and 35 ± 7% at 7 days post-injury (p = 0.0011) while LDH activity between injured and contralateral hemispheres remained unchanged after injury at every time points (p > 0.1769).

### The HP [1-^13^C] lactate-to-pyruvate ratio is sensitive to modulation of microglia/macrophages status following TBI

Next, our goal was to evaluate whether ^13^C MRSI of HP [1-^13^C] pyruvate can detect changes associated with the neuroinflammatory processes following injury.

We specifically narrowed down our study to 7 days post-injury as it corresponds to a subacute time point characterized by a high level of pro-inflammatory microglia and macrophages at the lesion site^5,34^. In order to evaluate possible direct and indirect contribution of microglia and macrophages to the increased HP [1-^13^C] lactate to pyruvate production observed after CCI, we used a CSF1R inhibitor (PLX5622) supplemented diet to deplete the brain resident microglial population and peripherally derived macrophages^29,35^.

**Figure 3:**
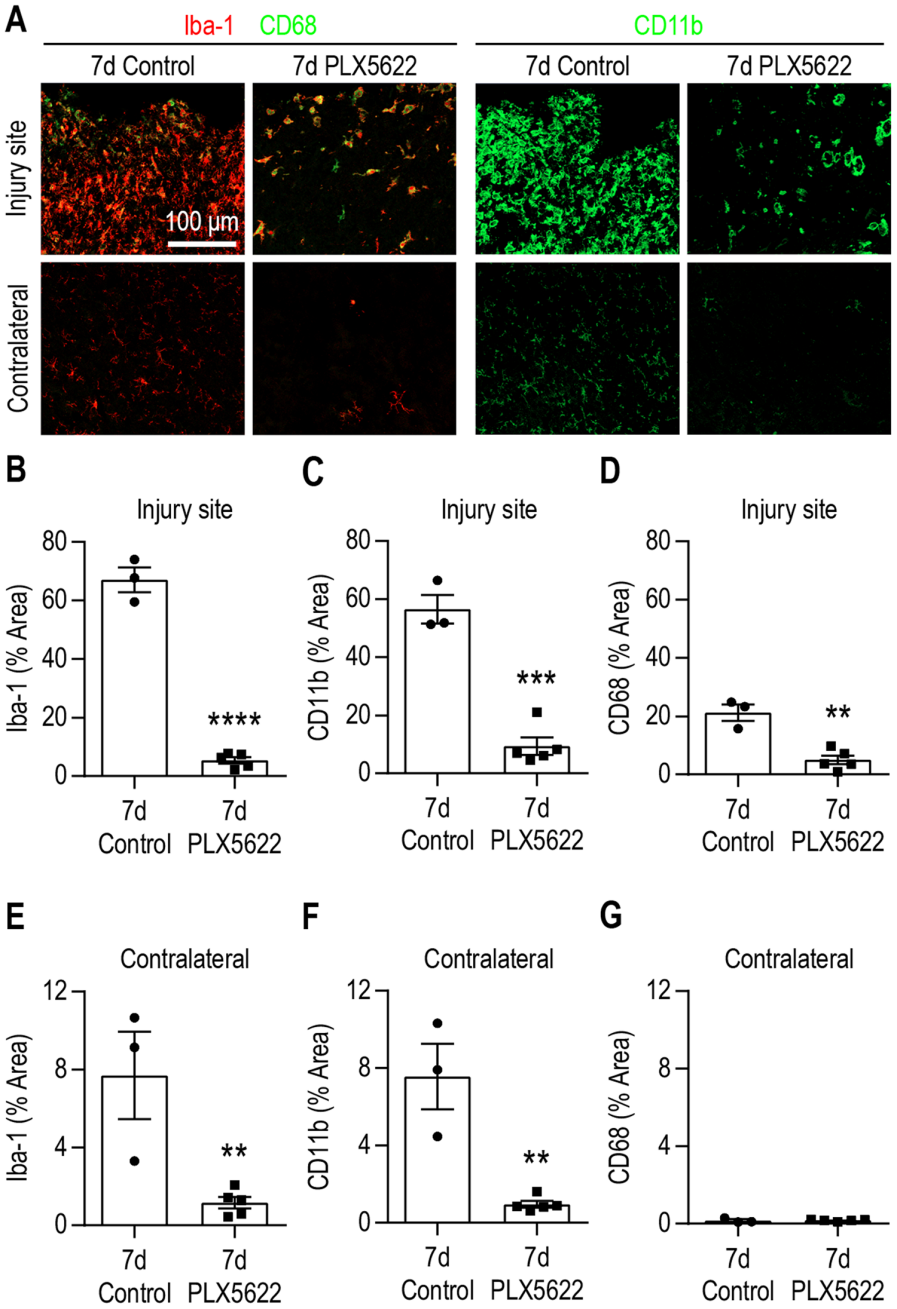
Immunofluorescence analyses following TBI and PLX5622 treatment. (**A**) Representative immunofluorescence images taken at 7 d post-injury showing a strong reduction of microglia/macrophages (Iba-1, red; CD11b, green), and CD68 expressing microglia/macrophages (yellow) in the group that received the PLX5622 compared to control diet. Quantitative analyses in the injured cortex confirmed decrease of (**B**) Iba-1 expressing microglia/macrophage population (92 ± 7%, p < 0.0001), (**C**) CD11b expressing microglia/macrophages (83 ± 5%, p = 0.0001), (**D**) the lysosomal marker CD68 (76 ± 4%, p = 0.0014), in the group that received the PLX5622 diet. Similarly, quantitative analyses in the contralateral cortex showed decrease of (**E**) Iba-1 and (**F**) CD11b expressing microglia/macrophage population (84 ± 7%, p < 0.0082 and 87 ± 6%, p < 0.0019, respectively) for mice that received PLX5622 compared to control diet, while the (**G**) lysosomal marker CD68 levels were below detection. All values are reported as mean ± sem (n = 3–5 mice per group).

We first verified that PLX5622 diet depleted microglia/macrophage in the brain at 7 days post-injury. Quantitative analyses of immunofluorescence staining presented in Fig. 3A–D showed a reduction of the total microglia and macrophages population (Iba-1, 92 ± 7% decrease, p < 0.0001; CD11b, 83 ± 5% decrease, p = 0.0001) as well as a reduction of the lysosomal marker CD68 (76 ± 4% decrease, p = 0.0014) at the site of injury in mice that received PLX5622 diet compared to mice who received the control diet. In the contralateral hemisphere, Fig. 3A and Fig. 3E–G, we observed a similar decrease in the population of microglia and macrophages (Iba-1, 84 ± 7% decrease, p = 0.0082; CD11b, 87 ± 6% decrease, p = 0.0019) while CD68 levels were below detection. Similar results were obtained in the hilar region of the hippocampus (Supplementary Fig. 4).

**Figure 4:**
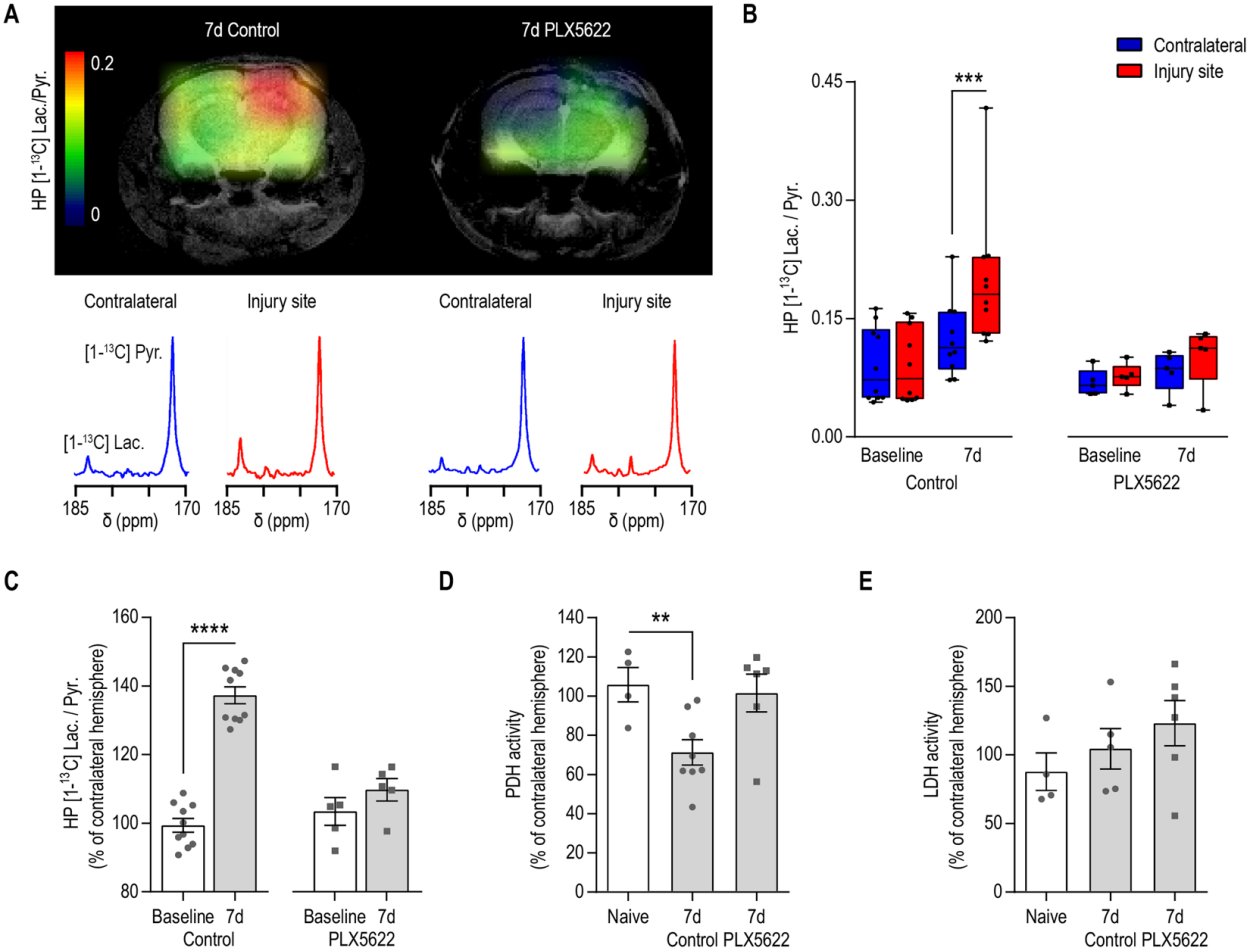
The HP [1-^13^C] lactate-to-pyruvate ratio is sensitive to modulation of microglia/macrophages status following TBI. (**A**) Representative heatmaps of the HP [1-^13^C] lactate-to-pyruvate ratios and corresponding HP ^13^C spectra showing increased HP [1-^13^C] lactate-to-pyruvate ratio and increased HP [1-^13^C] lactate production at 7d post-injury in the mouse that received the control diet but not in the mouse that received the PLX5622 diet. (**B**) Quantitative analyses of the HP [1-^13^C] lactate-to-pyruvate ratios revealed no significant differences between the injured and contralateral hemispheres of microglia depleted mice (Two-Way ANOVA, p = 0.089 for hemisphere effect, p = 0.1804 for time effect, p = 0.2792 for hemisphere and time interaction) at 7 days post-injury, contrasting with the increase of HP [1-^13^C] lactate-to-pyruvate ratio observed in mice that received the control diet (p = 0.0001). (**C**) HP [1-^13^C] lactate-to-pyruvate ratio, expressed as a percent change of the contralateral hemisphere, showed no change of the HP [1-^13^C] lactate-to-pyruvate ratio at 7 d post-injury compared to Baseline in mice that received the PLX5622, in contrast with the 37 ± 2% at 7 d (p < 0.0001) in mice that received the control diet. (**D**) PDH activity was decreased in the injured hemisphere of mice that received a control diet (p = 0.0011) but not in mice that received PLX5622 diet (p = 0.9429). (**E**) LDH activity remained unchanged (p = 0.1769). All values are reported as mean ± sem (n = 5–10 mice per group).

We then investigated whether T_2_-weighted MRI and ^13^C MRSI of HP [1-^13^C] pyruvate would be able to detect changes associated with the depletion of microglia/macrophages in mice that received the PLX5622 diet. T_2_-weighted MRI revealed the presence of a lesion and a cavitation in all microglia depleted animals 7 days after injury (Supplementary Fig. 5). As shown in Fig. 4A–C, a significant difference in the production of HP [1-^13^C] lactate can be observed between PLX5622 and control-treated mice (Figs 2 and 4A–C). Quantitative analyses confirmed that the HP [1-^13^C] lactate-to-pyruvate ratio at 7 days post-injury was not significantly different between injured and contralateral hemispheres in animals that received PLX6522 diet (Two-Way ANOVA, p = 0.089 for hemisphere effect, p = 0.1804 for time effect, p = 0.2792 for hemisphere and time interaction, Fig. 4B), contrasting with the increase of HP [1-^13^C] lactate-to-pyruvate ratio observed in mice that received a control diet (p = 0.0001 at 7 days post-injury, Figs 2C and 4B). Furthermore, as shown in Fig. 4C, no significant changes in the HP [1-^13^C] lactate-to-pyruvate ratio, expressed as percent increase of the contralateral hemisphere, could be detected between Baseline and 7 days following PLX5622 diet (p = 0.2138), contrasting with the 37 ± 2% at 7 days (p < 0.0001) in control-treated mice (Figs 2E and 4C).

In line with these findings, we did not detect a decrease in PDH activity in the injured hemisphere of mice that received a PLX5622 diet (p = 0.9429, Fig. 4D). LDH activity remained unchanged between injured and contralateral hemispheres for all groups (p = 0.1769, Fig. 4E).

## Discussion

In this study, we evaluated for the first time the potential of an innovative neuroimaging method, HP ^13^C MRSI, to monitor longitudinal changes in energetic metabolism in a CCI mouse model of TBI. We were able to report a significant increase in HP [1-^13^C] lactate-to-pyruvate ratio at acute and subacute time points following injury, which was associated with a decrease of PDH activity in the injured cortex. We further demonstrated that microglial depletion prior to CCI prevented the injury-induced increase of the HP [1-^13^C] lactate-to-pyruvate ratio at 7 days post-injury.

The present results are the first to use HP ^13^C MRSI to study the longitudinal modulations of the HP [1-^13^C] pyruvate-to-lactate conversion in the mouse brain after focal contusion injury. While DeVience *et al*. have previously demonstrated that HP ^13^C MRSI can detect acute metabolic alterations in a rat model of CCI^27^, numerous differences can be noted with the current study. Our study expands findings by investigating subacute and chronic end points following TBI. At acute time points, computed tomography (CT) is the method of choice because of its accessibility, speed of acquisition and accuracy in detecting skull fractures and intracranial hemorrhages that require urgent surgical intervention^36,37^. DeVience *et al*. used a lower field strength (3 tesla) with a single time point acquisition scheme (acquisition of a single spectra at 30 seconds post-injection of HP [1-^13^C] pyruvate), while we evaluated the dynamic conversion of HP [1-^13^C] pyruvate into lactate by acquiring a series of spectra every 3 seconds for a period of a minute. While our experimental design provides an intrinsically lower signal-to-noise-ratio of metabolites (due to higher field strength and smaller brain size of mouse versus rat), our dynamic acquisition scheme provides a more accurate evaluation of HP [1-^13^C] pyruvate-to-lactate dynamics and subsequent ratios values, which are not sensitive to user-induced experimental variations^31^.

Elevated lactate levels after TBI have been previously detected using ^1^H-MRS both in preclinical and clinical studies^8,17,20^. Because ^1^H MRS provides a measure of the steady-state levels of metabolites (intracellular + extracellular), increased lactate levels, as detected by this method, are often a combination of: 1)modulated intracellular lactate production through changes in enzyme activities, 2) accumulation of extracellular lactate originating from necrotic tissue, and/or 3) reduced lactate clearance rate. In contrast, the HP [1-^13^C] lactate-to-pyruvate ratios as measured by HP ^13^C MRSI reflect isotopic exchange from HP [1-^13^C] pyruvate to HP [1-^13^C] lactate through LDH, and are thus a combined indicator of the activity of enzymes that control the fate of pyruvate, and intracellular lactate pool-size^27,38^, as reviewed by Daniels *et al*.^31^. Before HP ^13^C MRSI, *in vivo* measurements of lactate-to-pyruvate ratios were previously accessible only by invasive procedures such as cerebral microdialysis^11^. In contrast to HP ^13^C MRSI which provide a readout of both enzymatic activity and intracellular lactate levels, microdialysis provides measurement of extracellular steady-state lactate-to-pyruvate ratios. It is well-established that extracellular lactate increase significantly following TBI and is associated with poor neurological outcome^8^, but whether or not prognosis is correlated with intracellular lactate is unknown. Hyperpolarized ^13^C MRSI and microdialysis thus appear as complementary strategies likely to provide distinct metabolic information. Future studies combining hyperpolarized ^13^C MRSI with microdialysis could be of interest as they would provide a more integrated picture of the cerebral metabolic state after injury.

Current neuroimaging methods used for the evaluation of TBI enable the detection of global anatomical alterations such as cavitation, hemorrhages, micro bleeds, hematomas, edema, grey and white matter shears, and diffuse axonal injury^36,37^. In our study, we observed discrepancies between anatomical alterations, particularly lesion size as detected by T_2_-weighted imaging, and HP [1-^13^C] lactate-to-pyruvate ratios. At 12 and 24 hours post-injury, despite similar lesion size, we observed a twenty-three percent increase in the HP [1-^13^C] lactate-to-pyruvate between the two time points. Similarly, the HP [1-^13^C] lactate-to-pyruvate ratios at 7 days were comparable to 24 hours post-injury, although there was a forty-six percent decrease in lesion size. Altogether, these results highlight the fact that anatomical and metabolic changes provide distinct information on the dynamic evolution of the injured tissue following TBI.

LDH is the enzyme that converts pyruvate into lactate and is overexpressed in most cancers^25,26^, and PDH is the enzymatic complex that controls the entrance of pyruvate into the TCA cycle. We therefore studied LDH and PDH levels at the same time points at which we measured metabolic changes. Unlike in cancer, no alterations of LDH activity were detected at any time points following injury. In contrast, we observed alterations of PDH activity following TBI, which likely reflect impairment of the oxidative properties of mitochondria previously observed^9^. Such decrease in PDH activity prevents pyruvate from entering the TCA cycle, which should subsequently result in an increased flux of HP [1-^13^C] pyruvate towards lactate production. In line with these reports, we measured a decreased PDH activity at 24 hours and 7 days post injury and demonstrated that these time points correspond to the highest HP [1-^13^C] lactate-to-pyruvate ratio values. Interestingly, at 12 hours post-injury, the measured HP [1-^13^C] lactate-to-pyruvate ratio was variable between individuals. At this time point, PDH activity in the injured cortex was not significantly lower than in the contralateral cortex, but displayed a trend toward decreased activity compared to Naïve animals. At 28 days post-injury, the HP [1-^13^C] lactate-to-pyruvate ratio were strongly decreased compared to 7 days post-injury (Fig. 2C) and PDH activity is no longer significantly different between injured and contralateral cortices.

Besides the initial mechanical insult caused to the brain at the time of injury, the secondary inflammatory responses that persist for days to weeks after injury can contribute to the neurological deficits observed after TBI. Interestingly, recent studies including these from our group, have shown that modulation of neuroinflammation following injury ameliorates long-term deficits and as such may represent a valuable tool for prevention of neurological disorders associated with TBI^1,3,34,39^. Elevated numbers of activated microglia and macrophages are present in the injured region following trauma, particularly within 3 days to 7 days post-injury^4,6^. Imaging studies using positron emission tomography (PET) and the translocator protein (TSPO) marker for neuroinflammation showed the highest levels of TSPO at 6 days post-injury^40^, in agreement with our findings of highest levels of HP [1-^13^C] lactate-to-pyruvate ratios at 7 days post-injury (Fig. 2C).

It is well-established that upon activation towards a pro-inflammatory phenotype, microglia and macrophages undergo metabolic reprogramming^41,42^ that results in increased glycolysis, lactate production, and inhibition PDH activity^43,44^. Consequently, the presence of pro-inflammatory microglia and macrophages may contribute to the observed increase of HP [1-^13^C] lactate-to-pyruvate ratios. In order to evaluate more selectively the metabolic changes associated with TBI-induced activation of microglia and macrophages, we used the CSF1R inhibitor PLX5622 to deplete the microglial and macrophage populations prior to and following CCI. In microglia depleted animals, we found that HP [1-^13^C] lactate-to-pyruvate ratios did not significantly increase in the injured hemisphere after TBI as compared to the contralateral hemisphere and that neither PDH nor LDH activity were significantly different between hemispheres. These results suggest a crucial role of microglia/macrophages in the elevated HP [1-^13^C] pyruvate-to-lactate conversion at 7 days post-injury. Such changes can reflect a lower number of pro-inflammatory microglia at the lesion site, as well as energy metabolism alterations of the injured tissue as a whole, as a consequence of altered immune environment. Notably, in this study, we used PLX5622 treatment solely as a tool to deplete microglia at the time of injury, in order to determine the specificity of the HP [1-^13^C] lactate-to-pyruvate signal. However, given the role of microglia in the initiation of the inflammatory response post-TBI^34,45^, it is conceivable to consider the therapeutic potential of PLX5622. A limitation to its therapeutic use is due to the fact that the initial inflammatory response after CCI takes place in the first 24-48 hours^34,45^ and PLX5622 takes 3–5 days to fully deplete microglia following CCI^30,35^. Furthermore, our study shows that the microglia depleted animals had a bigger lesion compared to control treated animals at 7 days post-injury, thus questioning a beneficial role of microglia at the time of injury (Supplementary Fig. 5). At this time, it is thus not clear if PLX5622 could have therapeutic potential for TBI, and more studies are needed.

In conclusion, our studies show that metabolic imaging using ^13^C MRSI of HP [1-^13^C] pyruvate can probe acute and subacute energetic disturbances following TBI. Importantly, we report that elevated HP [1-^13^C] lactate-to-pyruvate ratios at subacute time points are associated with the increased presence of microglia and macrophages. Altogether, these findings demonstrate that HP ^13^C MRSI has potential to enhance the diagnosis and monitoring of energetic imbalance following TBI and could serve a novel tool to examine altered immune response in other neurological disorders.

## Electronic supplementary material

Supplementary Information
