# Supplementary material for: In vivo metabolic imaging of Traumatic Brain Injury
Source: Sci Rep. 2017 Dec 13;7:17525. doi: 10.1038/s41598-017-17758-4 (PMC5727520; doi:10.1038/s41598-017-17758-4)
Supplement: Supplementary file 1 — Supplementary Information [file 41598_2017_17758_MOESM1_ESM.pdf]

SUPPLEMENTARY INFORMATION

In vivo metabolic imaging of Traumatic Brain Injury

Caroline Guglielmetti[1,2], Austin Chou[1,3], Karen Krukowski[1,3], Chloe Najac[2], Xi Feng[1,3], Lara-Kirstie Riparip[1,3], Susanna Rosi[1,3,4, 5, 6]*, Myriam M. Chaumeil[1,2] *

[1] Department of Physical Therapy and Rehabilitation Science, University of California, San Francisco, CA, USA.

[2] Surbeck Laboratory of Advanced Imaging, Department of Radiology and Biomedical Imaging, University of California, San Francisco, CA, United States

[3] Brain and Spinal Injury Center, University of California, 1001 Potrero Ave, Bldg. 1, Room 101, San Francisco, CA, 94110, USA.

[4] Department of Neurological Surgery, University of California, San Francisco, CA, USA.

[5] Weill Institute for Neuroscience, University of California San Francisco, CA, USA.

[6] Kavli Institute of Fundamental Neuroscience, University of California San Francisco, CA, USA.

Supplementary Figure 1: Experimental outline and HP ^{13}C MRSI dataset.

(A) Mice (n=10 CCI, n=5 Sham) underwent longitudinal MR imaging, prior to surgery (Baseline) and at twelve hours (12h), twenty-four hours (24h), seven days (7d) and twenty-eight days (28d) post-injury. A separate group of mice that did not undergo MR imaging was used for enzyme activity assays (n=4-8 per time point). **(B)** Mice (n=11) received PLX5622 or control diet for a total period of fourteen days. After exactly seven days of receiving PLX5622 or control diet, all mice underwent CCI surgery. A subset of these mice (n=5) underwent MR imaging one day prior to CCI (Baseline) and seven days post-injury. At seven days post-injury, mice were euthanized and tissue was collected for immunofluorescence analyses (n=5) and enzyme activty assays (n=6). **(C)** T_2-weighted MR image of a mouse head is overlaid with the voxels used for HP ^{13}C MRSI acquisition. The blue and red voxels contain the contralateral and injured cortices, respectively. Corresponding stack plot of HP ^{13}C spectra show HP [1-^{13}C] pyruvate delivery and subsequent HP [1-^{13}C] lactate production over time in the voxel of interest (3sec temporal resolution, 16 timepoints (TP)). All dynamic spectra (shown in black) are summed (shown in blue for the contralateral hemisphere, red for the injured hemisphere) and HP [1-^{13}C] pyruvate and HP [1-^{13}C] lactate levels were calculated for each imaging session and each animal.

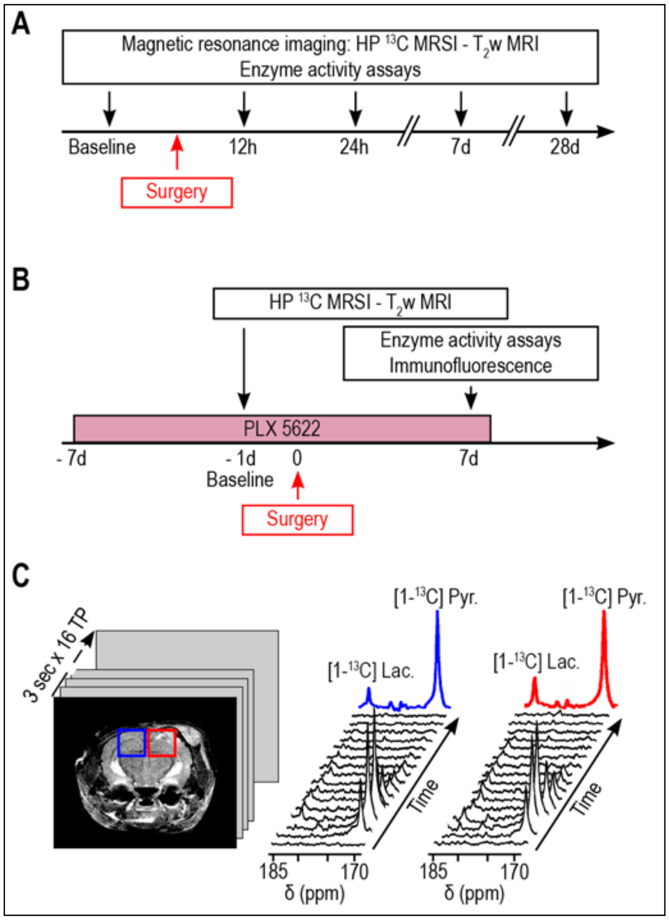

Supplementary Figure 2: Delineation of CCI-induced injury and ventricles

Representative delineation of the lesioned area (red), cavitation (blue) and ventricles (yellow) at 7 days post-injury on T_2-weighted images covering the whole brain.

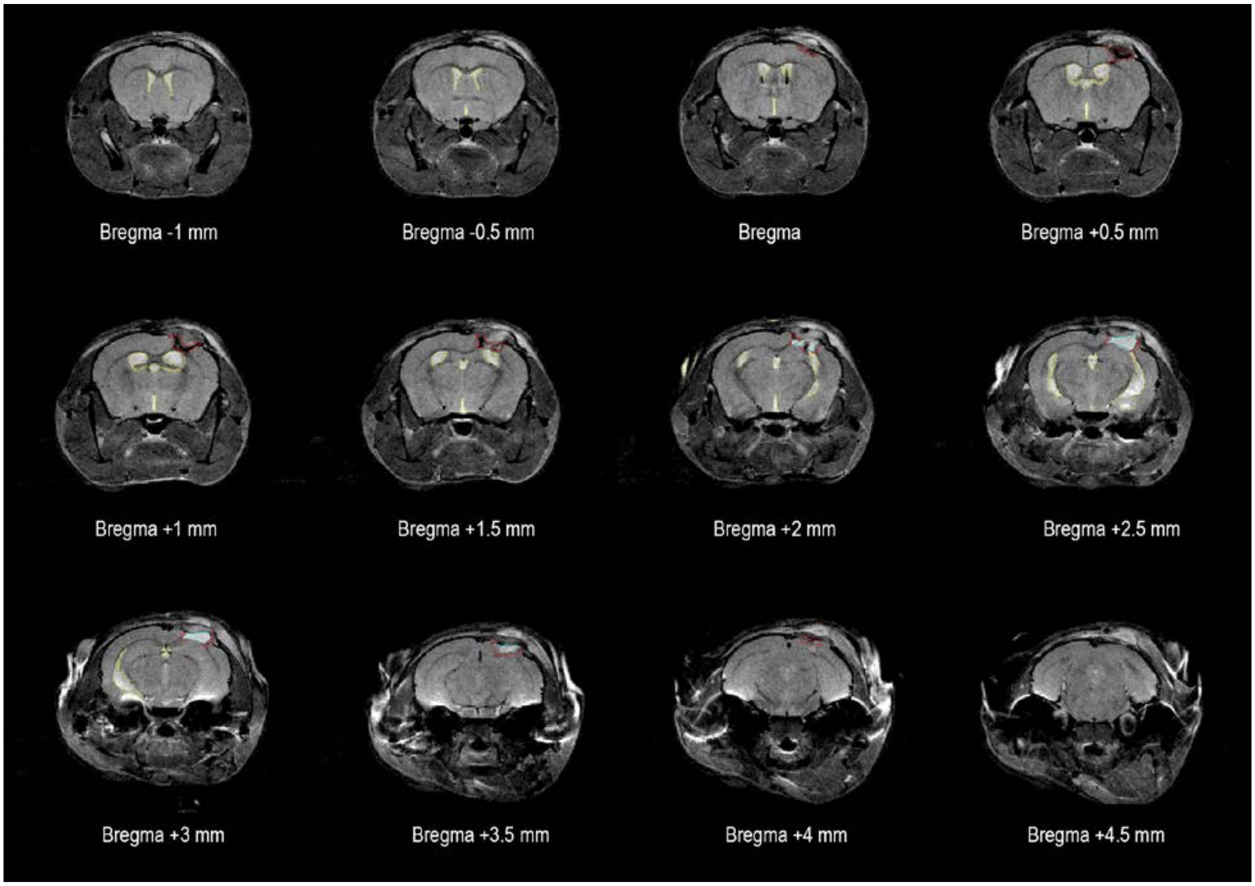

Supplementary Figure 3: The HP [1-^{13}C] lactate-to-pyruvate ratio is higher in CCI animals than in Sham animals.

HP [1-^{13}C] lactate-to-pyruvate ratios, expressed as percent change of the contralateral hemisphere, were significantly different between CCI (red, n=10) and Sham (green, n=5) animals (Two-Way ANOVA, $p=0.0001$ for group effect, $p<0.0001$ for time effect, $p=0.0702$ for group and time interaction). Specifically, the HP [1-^{13}C] lactate-to-pyruvate ratio of the CCI group was significantly higher compared to the Sham animals at 12 hours ($p=0.0456$), 24 hours ($p=0.002$) and 7 days ($p=0.0103$) post-injury but not at 28 days post-injury ($p=0.2861$). Additionally, whereas HP [1-^{13}C] lactate-to-pyruvate ratios overtime showed a significant increase in the CCI group, at 12 hours ($p=0.0004$), 24 hours ($p<0.0001$) and 7 days ($p<0.0001$) compared to Baseline, this ratio showed no significant difference at any time points in the Sham group ($p\geq0.1004$). All values are reported as mean ± sem.

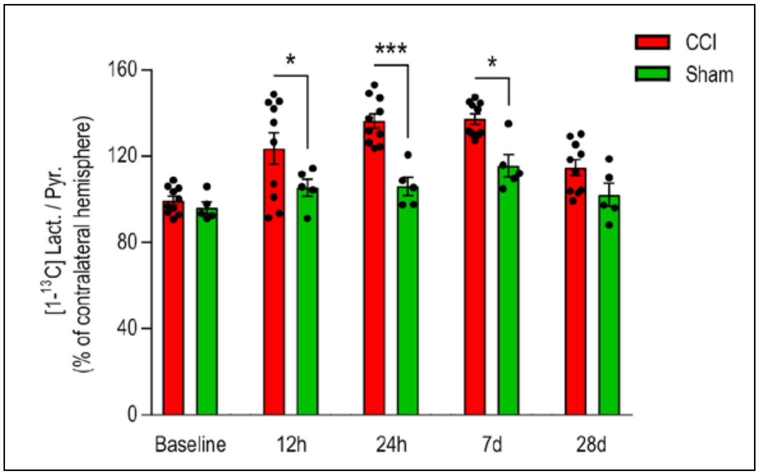

Supplementary Figure 4: Immunofluorescence analyses of the hilar region of the hippocampus following TBI and PLX5622 treatment.

(A) Representative immunofluorescence images of the hilar region of the hippocampus from the injured hemisphere at 7 days post-injury in mice that received fourteen days of PLX5622 or control diet. Quantitative analyses showed a reduction of **(B)** microglia/macrophages (Iba-1, red, p=0.0132) and **(C)** CD68 lysosomal marker (green, p=0.0139) in the group that received the PLX5622 compared to control diet, in the hilar region of the injured hemisphere. All values are reported as mean ± sem (n=3-5 mice per group).

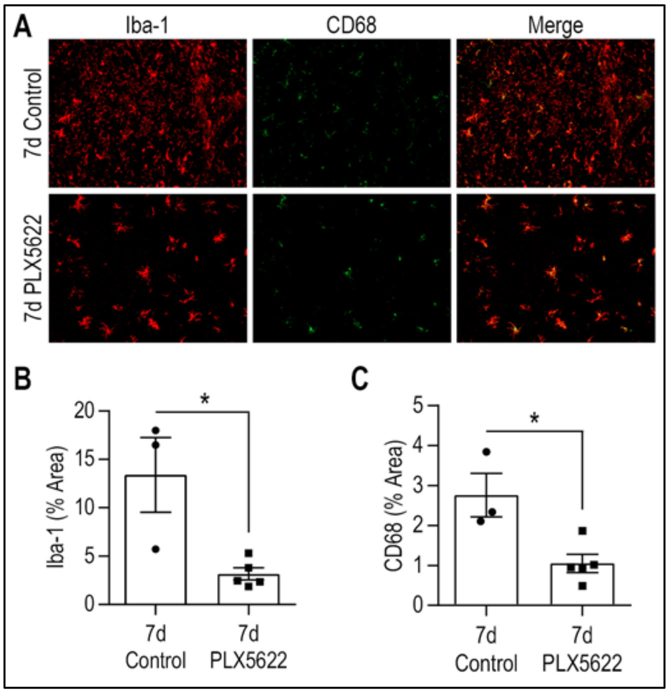

Supplementary Figure 5: T_2-weighted MR imaging of PLX5622-treated animals.

Quantitative analyses revealed a high lesion size at 7 days post CCI in PLX5622-treated animals ($p<0.0001$, compared to Baseline). The formation of a cavitation can also be observed at 7 days post-injury in these mice ($p=0.0041$, compared to Baseline). All values are reported as mean ± sem (n=11 mice).

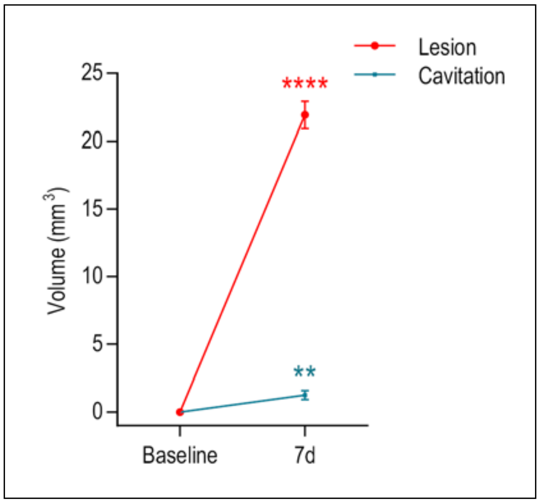

Supplementary table 1: Overview of number of animals used for each experimental procedure for the CCI and Sham control diet studies

		Baseline	12h	24h	7d	28d
CCI<br>Control diet	HP ^{13}C MRSI<br>T_2-weighted MRI	n=5 HP ^{13}C MRSI and T_2-weighted MRI<br>n=5 HP ^{13}C MRSI only				
	PDH and LDH activity	n=4 PDH and LDH	n=4 PDH and LDH	n=4 PDH and LDH<br>n=1 PDH only	n=5 PDH and LDH<br>n=3 PDH only	n=7 PDH and LDH
	Immunofluorescence	/	/	/	n=3	/
Sham<br>Control diet	HP ^{13}C MRSI	n=5 HP ^{13}C MRSI				

Supplementary table 2: Overview of number of animals used for each experimental procedure for the CCI PLX5622-treated study

		Baseline	7d
CCI<br>PLX5622 diet	HP ^{13}C MRS<br>T_2-weighted MRI	n=5 HP ^{13}C MRSI and T_2-weighted MRI<br>n=6 T_2-weighted MRI only	
	PDH and LDH activity	/	n=6* PDH and LDH
	Immunofluorescence	/	n=5*

* The same 11 animals were used for both the imaging and the *ex vivo* assays.
